# Opportunistic diagnosis of osteoporosis, fragile bone strength and vertebral fractures from routine CT scans; a review of approved technology systems and pathways to implementation

**DOI:** 10.1177/1759720X211024029

**Published:** 2021-07-10

**Authors:** Veena Aggarwal, Christina Maslen, Richard L. Abel, Pinaki Bhattacharya, Paul A Bromiley, Emma M. Clark, Juliet E. Compston, Nicola Crabtree, Jennifer S. Gregory, Eleni P. Kariki, Nicholas C. Harvey, Kate A. Ward, Kenneth E. S. Poole

**Affiliations:** Kingston Hospital NHS Foundation Trust, Kingston Upon Thames, UK; Health Evidence Matters, Bristol, UK; Imperial College London, London, UK; The University of Sheffield, Sheffield, UK; The University of Manchester, Manchester, UK; University of Bristol, Bristol, UK; Department of Medicine, UK; Birmingham Women’s and Children’s NHS Foundation Trust, Birmingham, UK; University of Aberdeen School of Medicine Medical Sciences and Nutrition, Aberdeen, UK; The University of Manchester, Manchester, UK; University of Southampton Faculty of Medicine, Southampton, UK; University of Southampton, Southampton, Hampshire, UK; University of Cambridge School of Clinical Medicine, Addenbrooke’s Hospital, NIHR Cambridge Biomedical Research Centre, Cambridge, CB2 0QQ, UK

**Keywords:** artificial intelligence, computed tomography, epidemiology, fragility fracture, innovation, Osteoporosis, QCT, screening, technology, vertebral fracture

## Abstract

Osteoporosis causes bones to become weak, porous and fracture more easily. While a vertebral fracture is the archetypal fracture of osteoporosis, it is also the most difficult to diagnose clinically. Patients often suffer further spine or other fractures, deformity, height loss and pain before diagnosis. There were an estimated 520,000 fragility fractures in the United Kingdom (UK) in 2017 (costing £4.5 billion), a figure set to increase 30% by 2030. One way to improve both vertebral fracture identification and the diagnosis of osteoporosis is to assess a patient’s spine or hips during routine computed tomography (CT) scans. Patients attend routine CT for diagnosis and monitoring of various medical conditions, but the skeleton can be overlooked as radiologists concentrate on the primary reason for scanning. More than half a million CT scans done each year in the National Health Service (NHS) could potentially be screened for osteoporosis (increasing 5% annually). If CT-based screening became embedded in practice, then the technique could have a positive clinical impact in the identification of fragility fracture and/or low bone density. Several companies have developed software methods to diagnose osteoporosis/fragile bone strength and/or identify vertebral fractures in CT datasets, using various methods that include image processing, computational modelling, artificial intelligence and biomechanical engineering concepts. Technology to evaluate Hounsfield units is used to calculate bone density, but not necessarily bone strength. In this rapid evidence review, we summarise the current literature underpinning approved technologies for opportunistic screening of routine CT images to identify fractures, bone density or strength information. We highlight how other new software technologies have become embedded in NHS clinical practice (having overcome barriers to implementation) and highlight how the novel osteoporosis technologies could follow suit. We define the key unanswered questions where further research is needed to enable the adoption of these technologies for maximal patient benefit.

## Introduction

**Figure 1.**
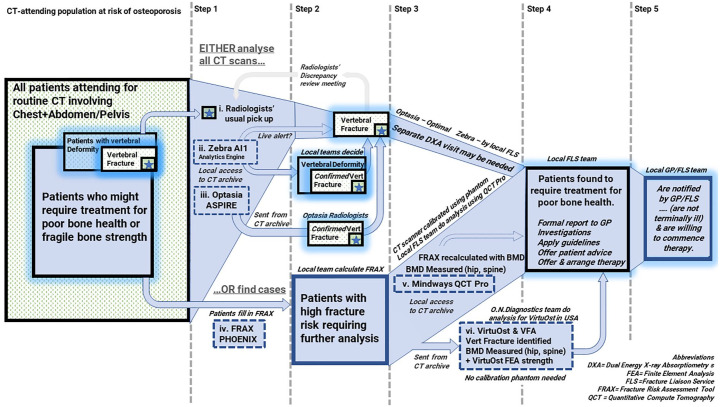
Comparison of available products and services (i–vi) to measure bone health in the CT-attending population, their place in screening and the barriers to adoption in a health service (dashed grey horizontal lines). A large proportion of older patients have previously undiagnosed osteoporosis (left panel), and some even have previously undiagnosed vertebral fractures (with or without osteoporosis). Starting with all older patients attending for routine CT, there are tools to screen all scans (Optasia ASPIRE and Zebra AI1) to identify possible vertebral fractures. Other tools (Mindways QCT Pro and VirtuOst) are best suited to some form of fracture risk assessment, with higher-risk individual scans being selected for analysis of density, strength and vertebral fracture (depending on the system). CT, computed tomography; DXA, dual energy X-ray absorptiometry; GP, general practitioner; FEA, finite element analysis; FLS, fracture liaison service; FRAX, fracture risk assessment tool; QCT, quantitative CT.

With modern computed tomography (CT) scans, some portion of the patients’ spine is visualised in detail during ordinary chest, abdomen and pelvis scanning, giving ample opportunity for diagnosing osteoporosis and for various methods of vertebral fracture assessment (VFA) technologies. These range from manual identification right through to semi-automated and fully automated methods, some of which are accepted for diagnosis by international specialist societies. A summary of products and services available to measure bone health in the CT-attending population is provided in Figure 1, highlighting the niches they occupy in typical primary and secondary osteoporosis screening strategies. This review focus not only on the technologies, but also on the barriers to their adoption.

Artificial intelligence (AI), along with its sub-disciplines of machine learning (ML) and deep learning (DL) are emerging as key technologies with the potential to improve patient outcomes. ML is a set of software algorithms and statistical models used to perform a specific task, without using explicit instructions. This approach is different from the other types of software we review, where products have emerged from coding done intentionally (based on what developers already know about proven osteoporosis predictors). With AI, large data sets of CT images are coupled with knowledge of eventual fracture outcomes and prevalence to ‘learn’ which imaging features predict the outcome of interest.

This Rapid Review aims to provide a comprehensive review of the topic but is not a full systematic review of all related literature. Cochrane guidance on Rapid Review methodology was published recently (https://tinyurl.com/y6ce5g4v). For in-depth evaluation of the technical CT methodologies, we recommend two recent review papers.^1,2^

## Definitions and searches for the rapid evidence review

### Definition of ‘approved’ software and services

This review considers all technologies that have either received United States (US) Food and Drug Administration (FDA) approval, ISO 13485 certification (in the case of Medical Devices that involve a phantom), a European CE mark for diagnosis, or are National Health Service (NHS) Care Quality Commission (CQC) regulated technology services. We also evaluated studies showing the cost effectiveness of the use of CT technologies. We consider each technology, its mechanism, integration within clinical systems and the evidence for its efficacy.

### Patient and public involvement

The Patient and Public team of the Royal Osteoporosis Society conducted a survey of members seeking their views on different research questions through their Bone Academy Patient Insight Group (December 2019–January 2020). In total, 2313 patient responders with osteoporosis (from 7237 mailed) graded priorities and expectations from 13 key areas across the domains of Osteoporosis Causes, Technology and Service Effectiveness. Opportunistic detection of osteoporosis and vertebral fractures from CT data was the highest ranking priority, with 70% of patients thinking that patients were ‘extremely likely to benefit’ from the idea and a further 22% ‘very likely to benefit’ (92% score in total).

### Data sources

Data sources searched include:

- NICE Evidence library portal;
- Systematic reviews via: Cochrane Library;
- Electronic bibliographic databases: Embase, Medline; Tripdatabase; Web of Science;
- Websites: NICE;
- Search engines: Google Scholar and Google;
- Theses and dissertations;
- Individual companies: Mindways; Optasia Medical; ON Diagnostics; Zebra Medical were all contacted for supporting research literature relevant to their technology.

Grey literature, such as research studies carried out by charities and research institutes, reports, commentaries and review papers from government, policy bodies and professional organisations, was reviewed in support of the academic literature. In particular, the Grand Challenge AI for Radiology engine for CT products relating to osteoporosis and fracture terms was reviewed.

### Search strategy

A Boolean search was performed using the operators AND, OR, NOT in combination with the following keywords, index headings and free text: Computed tomography; biomechanical computed tomography; computed axial tomography; computer assisted tomography; CT; computed tomography x-ray absorptiometry; CTXA; finite element analysis; FEA; dual energy X-ray absorptiometry; DXA; DEXA; osteoporosis; bone density; bone mineral density; fracture; screening; diagnosis; diagnostic; opportunistic; Mindways; Optasia; O.N. Diagnostics; Zebra Medical. Truncation techniques using asterisks and wildcard techniques using question marks were employed when free text searching. Additionally, reference lists of key relevant primary research, systematic reviews and meta- analyses and grey literature were examined to identify further studies. Citation searches of key relevant articles were undertaken. Targeted searches for publications by key academic researchers were made. Searches were limited to the English language.

## Osteoporosis, vertebral fragility fractures, fracture liaison services and case-finding during CT

### Osteoporosis and vertebral fragility fractures

Osteoporosis is a disease that causes bones to become weak and fragile. It is a major cause of disability, loss of quality of life and early death in the older population and poses a significant public health problem in a globally ageing population. The condition is usually asymptomatic until a fracture occurs, and patient perception of fracture risk is often underestimated.^3^ Vertebral fragility fractures occur either spontaneously, as a result of normal activities such as lifting or coughing, or from mild trauma. These spinal fractures are the most common of all osteoporotic fragility fractures, occurring in 25% of men and post-menopausal women.^4^ Under-diagnosis is a particular issue for vertebral fractures as only a minority result from a fall and symptoms may be attributed by both patients and clinicians to another cause.^5^ Nearly all fractures are associated with an increased risk of future fracture, regardless of age, bone mineral density (BMD) and fracture location.^6^ According to the International Osteoporosis Foundation (IOF), 520,000 fragility fractures occurred in 2017 in the United Kingdom (UK), costing £4.5 billion. This expenditure is set to increase by 30% by 2030 due to the ageing population.^7–10^

Treatment and behavioural interventions for people diagnosed with osteoporosis and vertebral fractures have been shown to reduce hip and other fracture rates by 40–70%.^11^ A recent comprehensive review has found that secondary prevention strategies appear to be better developed and more successful than primary prevention strategies.^12^ However, currently less than half of patients with a fragility fracture undergo secondary osteoporosis screening.^13^ This is a missed opportunity, since the ‘lowest hanging fruit’ in secondary prevention of fractures are those people presenting to secondary care with a first fragility fracture. This group includes people found to have an incidental vertebral fracture after a CT scan, which is the subject of this evidence review.

### The role of fracture liaison services

The failure to treat osteoporosis even after knowledge of a fragility fracture is known as the ‘osteoporosis treatment gap’. The percentage of women who did not receive treatment after a fracture was estimated by the IOF to be 49%.^9,10^ Despite proven efficacy of osteoporosis therapy, simple guidelines and multiple simple therapeutic options, treatment prescription rates remain sub-optimal.^14^ Clinical care systems have been slow to incorporate secondary prevention. The usual care following a low-trauma fracture (including hip and vertebral) can still lack a simple evaluation and/or treatment of the osteoporosis that contributed to the fracture.^15^ A multidisciplinary fracture liaison service (FLS) can facilitate case identification, investigation and intervention,^16^ reducing the osteoporosis treatment gap and preventing fractures.^17–20^ Their effectiveness at reducing the risk of subsequent fracture is supported by level 1 evidence from systematic reviews with meta-analyses.^21,22^ Currently, it is unusual for FLS to follow up patients whose vertebral fractures are identified ‘opportunistically’ during CT scanning for other reasons.

### Osteoporosis case-finding during CT

Patients attending hospital for routine CT are a group of patients who might be suitable for targeted case-finding. The Royal Osteoporosis Society and the Royal College of Radiologists (UK) have recently overseen education and audit initiatives focused on improving the identification of osteoporotic vertebral fractures in imaging done for other reasons, including CT. Their recent audit of UK radiology departments found that only 26% of vertebral fractures visualised incidentally on CT images were reported accurately, and less than 3% of patients were referred onwards for appropriate management (see Figure 1).

This review focusses on approved technology systems and their potential for adoption in health services, by which we mean systems that have received either FDA approval, ISO 13485 certification (in the case of Medical Devices that involve a phantom), a European CE mark for diagnosis, or are NHS CQC regulated technology services. This does not currently include the substantial body of research literature investigating the utility of direct Hounsfield unit (HU) estimation of bone health. In the UK fracture risk assessment is generally recommended by the National Institute of Health and Care Excellence (NICE) when patients have specified co-morbidities – a so-called targeted case-finding approach.^11,23^ In the case of patients attending hospital for routine CT, these patients will often fulfil the NICE criteria that recommend a fracture risk assessment due to age and co-morbidities. However, CT-attenders are not currently targeted for risk assessment. Fracture risk assessment using the online FRAX tool to input simple questionnaire answers gives a person-specific 10-year risk of osteoporotic fracture. A ‘FRAX 10-year risk’ of major osteoporotic or hip fracture is therefore the commonest method used to identify individuals at high risk of fracture in both primary and secondary prevention. The electronic output of FRAX in the UK is matched with nationally agreed osteoporosis guideline thresholds (National Osteoporosis Guideline Group: NOGG) that indicate the need for drug treatment. However, the current prevalence of high-risk patients (by FRAX) attending CT units is not known. Enthusiasm to screen all CT attenders with FRAX (or for that matter to investigate every single CT image for osteoporosis) must be tempered by the clear advice concerning primary osteoporosis screening programmes in the UK; the National Screening Committee (https://legacyscreening.phe.org.uk/osteoporosis) do not recommend population screening, citing a lack of effectiveness criteria. Nevertheless emerging data from the US on screening with CT bone strength analysis are encouraging.^24^

### Diagnosing osteoporosis using DXA

Dual energy X-ray absorptiometry (DXA) remains the traditional imaging technique in osteoporosis and gold standard for diagnosis. It uses an X-ray and detector system to measure the mineral content of bone and is especially well suited to the average lumbar spine (usually lumbar vertebrae 1 and 2 or 1, 2, 3 and 4) as well as the proximal femur [femoral neck (FN) and ‘total hip’]. The Word Health Organisation (WHO) definition of osteoporosis is based on a DXA measurement of BMD, deriving from evidence showing a clear link between lower BMD and increased fracture risk.^25^ Diagnostic criteria use standard deviation (SD) scores of BMD related to peak bone mass in healthy young women, with osteoporosis being defined as a BMD T score of −2.5 or less and low bone mass (osteopenia) as a BMD T-score between −1 and −2.5.^26^ DXA BMD values, particularly derived from the FN, are a very good indicator of future fracture risk and have long been incorporated into modern fracture risk estimating tools such as FRAX. DXA is subject to the limitations of a planar two-dimensional (2D) technology to represent a three-dimensional (3D) bone, and availability is patchy. Another limitation of DXA spine measurements are the inaccuracies in the setting of degenerative spinal pathology, and that this measurement is limited only to the lumbar spine. Each T-score unit decrease in BMD confers approximately a doubling of fracture risk. However, most osteoporotic fractures occur in individuals who do not have an ‘osteoporotic range’ BMD. In addition, other risk factors (e.g. age, sex, previous fracture) are associated with fracture risk independently of BMD.^27,28^ DXA has a very low radiation dose.

### Diagnosing osteoporosis and vertebral fractures using CT; technology and services overview

Quantitative CT (QCT) is an established 3D imaging technique having been used in clinical practice since the 1970s. For a clinician seeking a diagnosis of osteoporosis in their patient, a CT scan is requested far less frequently than DXA due to the higher radiation dose with CT. Spine QCT measures the true volumetric BMD (vBMD) of trabecular bone within vertebrae, usually the average of lumbar vertebrae 1 and 2 (or 1, 2 and 3) with low values being an excellent risk marker for prevalent vertebral fracture.^29–31^ QCT BMD measurements of the spine do not give the same values as DXA (QCT Pro Spine, Software Mindways, Austin, TX, USA and VirtuOst Spine, O.N. Diagnostics, Berkeley, CA, USA). Software can also ‘project’ areal BMD (aBMD) of the hip from CT scans, more akin to DXA imaging. Like DXA, this areal density (g/cm^2^) is measured at the ‘FN’ and ‘total hip’ regions (QCT Pro CTXA and VirtuOst Hip) – the measurements are directly comparable with DXA and can be entered specifically into the FRAX online tool to give patient-specific 10-year fracture risk and UK treatment thresholds using either the ‘Mindways QCT’ drop-down option (CTXA) or the ‘T-score’ option (VirtuOst).

Developed as an adjunct to DXA and performed on the same machine, ‘vertebral morphometry’ images [also called VFA] are used to visualise a patient’s entire lateral spine encompassing key areas at risk of fracture, from the thoracic to the lumbosacral region. Fractures are diagnosed by reference to various shape criteria in the lateral projection of the wedged, crushed or biconcave-appearing vertebra. Software technology-based services are now emerging that automate or semi-automate the process of identifying vertebral fractures from CT data, where scans are done for other medical reasons and not for the primary purpose of osteoporosis assessment. In this emerging field, CT scans can be sent to an external company (ASPIRE service, Optasia Medical, Manchester, UK or AI1 Solutions using Zebra Bone Health algorithm, Zebra Medical Vision Ltd, Shefayim, Israel). In other scenarios, the automatic identification of vertebral fractures is integrated with other point of care AI tools visible to the radiologist reviewing the original CT scan (AI1 Integrations, using the Zebra Bone Health Algorithm).

## Opportunistic ancillary screening for osteoporosis, low bone strength and vertebral fractures in CT scans done for other indications

### Practical aspects of ancillary screening of CT data

According to the latest data from NHS England, almost 6 million CT scans were performed October 2019–October 2020 for patients in England.^32^ Of these, over 1 million were estimated to include the chest and/or abdomen. If opportunistic ancillary screening was performed on these CT scans, earlier treatment for those with previously undetected osteoporosis might have saved and improved lives, and potentially saved significant costs to healthcare systems. Opportunistic CT-based screening methods have the potential to be light-touch (in terms of cost, time and inconvenience to stakeholders) and to prevent unnecessary hospital visits and further irradiation.^33^

Academic researchers, software companies and service providers have realised the potential to diagnose osteoporosis and identify vertebral fractures as an ‘added extra’ service applied to CT scan images that have already been taken for other clinical reasons. A single clinical CT scan consists of a batch of hundreds of consecutive 2D slices (axial sections) through a person (the number of slices depending on the predetermined slice thickness and the amount of the body covered by the scan). The software and services for screening for osteoporosis and vertebral fractures are not usually installed on the radiographers’ CT scanning personal computer (PC) that drives the CT scanner. Instead the bone analysis software can be located either on nearby ‘standalone’ PCs or on the radiologists’ analysis terminal [called a picture archive and communication system (PACS) diagnostic workstation] or even at a totally distant site. In the latter case, analysis can be done by technical staff rather than radiographers, and sometimes away from the hospital, as long as the organisation providing the service is CQC-approved by the NHS. Using computer software to diagnose osteoporosis or vertebral fractures can be done any time; from minutes to hours after the patient has left the CT department, up to many months after the original scan. Here, the extra radiation dose is zero and the patient may be spared additional DXA imaging visits.

### Diagnosis and fracture-prediction from QCT imaging technologies; landmark studies, diagnostic criteria and regulatory aspects

Traditionally, QCT measurements of vBMD have been made with specialised software and the patient lying on an ergonomic, slim bone calibration phantom. Phantoms are manufactured with materials of known density, usually calcium/potassium hydroxyapatite and are placed under the patient’s lower back and hips in order to mitigate for the variability in CT scanners by converting CT attenuation (measured in HU) to vBMD.^34^ This is known as synchronous calibration. Bone density measurements made using synchronous calibration have been in clinical practice for many years and are usually reported by reference to the American College of Radiology criteria, where spine BMD values below 80 mg/cm^3^ are considered osteoporotic. Age- and sex-specific reference ranges of spine QCT BMD have long been available for adults and diagnostic test data are also published.^29,30^ While age-related reference ranges are used to generate Z-scores, to avoid confusion, T-scores (the diagnostic WHO criteria for DXA) are not generally used. A T-score is rather a DXA-specific concept and probably best kept linked to that particular planar, summative imaging method.

In 2014, a suitably designed and powered prospective study of healthy adult men and women was published confirming diagnostic accuracy of the 80 mg/cm^3^ threshold (i.e. ‘ACR standardised’ phantom-calibrated spine vBMD) in predicting (a) vertebral fracture (with complete 5-year follow-up spine imaging for coverage of all vertebral fractures occurring in the cohort) and (b) incident hip fracture (using ICD hospital codes). Average spine vBMD (L1 and L2) measured by QCT was highly statistically significantly associated with incident vertebral fracture; age-adjusted odds ratio (OR) for vertebral fracture was 3.1 [95% confidence interval (CI) 2.2–4.7] for every one SD lower spine vBMD, giving a typical 75-year old female or male with baseline vBMD of 80 mg/cm^3^ a 14.6% (11.1, 19.3) predicted probability of vertebral fracture.^31^ A clinical study recently found that spine QCT was superior to DXA in predicting incident vertebral fracture in clinical practice, but caution is needed when evaluating the study.^35^ Data from dedicated healthy ageing cohorts that match baseline high-quality CT imaging to contemporaneous modern DXA methods are needed.

More recent technological advances have opened the possibility of calculating the BMD of a patient without the phantom being present at the time of scan, known as ‘phantom-less’ or ‘asynchronous’ approaches. The various methods of achieving this are listed in Table 1. The application of this nascent technology is highlighted below.

**Table 1.** Summary of the approaches used for CT measurements of bone density.^24,26,36–41^

Method	Notes
Traditional phantom-based synchronous calibration	• Patient lies on an ergonomic phantom with materials of known densities (usually 2–5 rods of different human tissue density equivalents)
	• CT attenuation values of the hip or spine are converted to BMD by reference to the known density values (QCT Pro)
	• Hip scans can be adapted to derive areal BMD, suitable for use in FRAX (CTXA)
Phantom-less synchronous internal calibration	• No external calibration phantom scanned
	• CT attenuation of adjacent internal tissues (e.g. blood or fat) used to calibrate attenuation measurements (VirtuOst)
	• Can be adapted to derive areal BMD, suitable for use in FRAX (VirtuOst Hip, T-score)
Asynchronous external calibration	• Phantom scanned regularly.
	• Simple, single-material phantom (Mindways Model 4 phantom, CliniQCT)
	• Hounsfield numbers of bone are then compared with phantom
	• Asynchronous CT of proximal femur can be adapted to derive areal BMD, suitable for use in FRAX (CliniQCT CTXA)
Asynchronous external calibration with the ACRad phantom	• Routine calibration using ACRad phantom
	• Direct CT attenuation values (HUs) are used to determine trabecular radiodensity without a BMD-specific calibration phantom
	• Does not require specialised software – can be performed on PACS workstation or any computer with standard tools used for viewing CT images

ACRad, American College of Radiology; BMD, bone mineral density; CT, computed tomography; CTXA, CT X-ray absorptiometry; FRAX, fracture risk assessment tool; HU, Hounsfield units; PACS, picture archive and communication system; QCT, quantitative CT.

Commercially available methods can also identify individuals at high risk of fracture using CT combined with FEA.^1,42^ Initially introduced 40 years ago, FEA is a non-destructive computer simulation method that estimates the stiffness of a structure by dividing it into a number of simple parts, termed finite elements, that are connected by points termed nodes. Combinations of FEA and *in vivo* bone imaging data have significantly improved the estimation of bone mechanical behaviour compared with imaging alone.^43,44^ This combined ‘biomechanical CT’ (BCT, VirtuOst software, O.N. Diagnostics) approach provides non-invasive estimates of the breaking strength of the hip and spine. Combining that measurement with a CT-based measurement of a DXA-equivalent hip BMD T-score, BCT provides a more comprehensive diagnostic assessment of osteoporosis than bone strength or BMD alone.^36,45–47^ In the aforementioned diagnostic accuracy study, the age-adjusted BCT OR for incident vertebral fracture was 4.3 (2.4, 7.6). A 75-year old woman whose L1 vertebral strength lies exactly on the Fragile Bone Strength threshold for a female (4500N) has a predicted probability of vertebral of 22.2% (18.5, 26.4). The predicted probability of vertebral fracture increased more steeply with declining L1 Strength than for vBMD.^31^

From a regulatory perspective, the CQC is the UK’s independent regulator of health and social care. Their report from March 2020 highlighted a range of observations and recommendations.^48^ They emphasised the need for good governance of clinical, information, technical and human aspects of any ML tools in diagnostic services. They stated that most suppliers of ML applications in diagnostics will not need to register with CQC, only those that deliver clinical activity themselves. These few will need to be regulated and assessed by national standards to ensure safety and efficacy. The report emphasised that there is need for more assurance about the clinical aspects of algorithms in ML and clarity on how they can be implemented to ensure high-quality clinical care. There is also the need for technology suppliers to be clear what their products, solutions and devices do and how they perform, as suppliers do not always accurately state whether their products use ML, which makes it harder to implement devices safely.

## Clinical effectiveness of currently available tools and services to diagnose osteoporosis, low bone strength and vertebral fractures in CT scans done for other indications

### VirtuOst software, including FE (BCT)

VirtuOst fracture risk assessment service using strength-based classifications is referenced by the International Society of Clinical Densitometry (ISCD) guidelines as suitable for osteoporosis identification, fracture risk assessment and therapy monitoring. This technology has been solely used in the Mayo Clinic (Rochester, MN, USA) for the last 4–5 years, but increased cover and reimbursement by Medicare (as an official screening test for osteoporosis) may lead to wider adoption. The patient’s CT scan is sent to the company electronically, the analysis performed by the company and the results sent back to the ordering physician. Regulatory approval anywhere outside the US has not yet been applied for.

VirtuOst identifies osteoporosis on the basis of BMD, bone strength measurement, or both, at the hip and spine using synchronous internal calibration (Table 1).^31,49^ The results are of diagnostic quality and do not need verification by DXA or any other tests. DXA-equivalent areal BMD T-scores are obtained for the FN, which can therefore be used with FRAX (using the ‘T-score’ drop-down box on the FRAX website).^50–53^ A typical report from VirtuOst has areal hip BMD (in g/cm^2^), the associated T-score, plus L1 vertebral volumetric BMD in mg/cm3, as well as a measure of strength of the hip and vertebra calculated from 3D FEA. The latter (measured in Newtons) is reported by reference to a threshold of ‘fragile bone strength’ against age-specific expected values. Finally, VirtuOst encompasses a VFA covering as much of the spine as is captured in an individual’s scan. Each of these components of the VirtuOst clinical report has been verified independently in large fracture prediction studies for hip and spine fractures so that a ‘high risk’ individual might achieve that through one or more components of their analysis.

The various components of the VirtuOst service were validated in nine fracture-outcome studies, mostly conducted in the US and Iceland. The BCT technique showed BMD scores obtained from DXA and CT colonography had a high degree of agreement (*R*^2^ = 0.84).^54^ In a cohort of 136 patients undergoing CT enterography (CTE), this technique also demonstrated a high degree of sensitivity and specificity for confirming osteoporosis (85.7% and 98.5%, respectively) or osteopenia (85.1% and 85.4%, respectively).^55^ In another cohort of 136 women undergoing CT enterography, BCT analysis identified osteoporosis (as defined by DXA) with 100% specificity in 8 out of 8 patients, and 98.4% specificity in 126 of 128 patients (95% CI: 94.5%, 99.6%).^56^ These data are further validated by a US retrospective case-cohort study of 4000 participants, in which accuracy of the BMD T-score as measured by VirtuOst analysis was consistent with DXA for all fracture-risk metrics and both sexes.^57^ Importantly, the use of VirtuOst could be vital in inflammatory bowel disease monitoring, where a study of 257 patients who underwent CTE and BCT showed 54.5% of patients had high/increased fracture risk, of which 40.3% did not meet any of the Cornerstone screening criteria (IBD checklist for monitoring and prevention in bone health).^58^

The prospective diagnostic accuracy of 2D measurements of FN BMD (using QCT) for incident hip fracture was established recently using the VirtuOst method.^31^ Average FN aBMD measured by QCT was highly statistically significantly associated with incident hip fracture; age-adjusted OR for incident hip fracture was 3.5 (2.5–5.0) for every one SD lower FN BMD using QCT, giving a 75-year old female with baseline FN BMD T-score of −2.5 a predicted probability of hip fracture of 21.8% (17.0, 27.5). For men these figures were slightly higher at OR 3.7 (2.5, 5.6) and a 33.4% (23.3, 45.4) probability of hip fracture.

### Mindways QCT Pro software (QCT Pro, CliniQCT, CTXA hip)

Mindways QCT software calibrates HU measured by any CT machine against a bone-density equivalent phantom to give consistent hip and spine bone density measurements across devices. There are two main products; QCT Pro and CliniQCT; the difference being that CliniQCT permits opportunistic osteoporosis hip and/or spine assessment from abdomino-pelvic scans in any CT scanner that has been calibrated with the supplied Model 4 cylinder phantom (a small cylinder of uniform material that calibrates using the CT beam hardening effect, monthly). CliniQCT is the system most relevant to this review. BMD measurements can be made from a wide range of scans such as abdomen/pelvis/spine CT, CT urography and cancer related positron emission tomography (PET-CT): the only requirements are that the scan covers an appropriate skeletal region. This requirement for a local user/analyst is one aspect that separates Mindways software from the other technologies in this review. There are clinical distributors in 12 countries spanning the US, Europe and Asia who assist with installation/training.^59^

CliniQCT involves the FDA-approved and ISO 13485-approved ‘asynchronous QCT calibration’ method to analyse bone density in CT images from any scanner that has been calibrated by the Model 4 cylinder phantom. This enables the opportunistic use of CT data sets acquired for other purposes that did not include a CT calibration phantom in the patient images; a technical obstacle that is also overcome (albeit using a different method) by VirtuOst. Like VirtuOst, Mindways’ DXA-equivalent CTXA hip module gives areal bone density values (in g/cm^2^) and T-scores that are approved for diagnosis by the ISCD, as well as 3D volumetric analysis of BMD in the spine (in g/cm^3^). Phantom scans maintain precision and account for drift. Mindways’ CTXA data analysed in both a conventional and asynchronous manner confirmed diagnostic accuracy with excellent intra-and inter-reader reliability and correlation with DXA (*r*^2^ = 0.907, *r*^2^ = 0.82).^38,60–63^ QCT Pro is different insofar it requires a Model 3 flattened, curved phantom to be placed under the patient during a dedicated hip and or spine scan, plus a separate QA phantom is fitted onto the Model 3 for monthly quality scans (or after a CT X-ray tube change).

Mindways software can be run on a standard PC and does not require radiology-specific monitors or computers. Unlike the other systems in section 4 of this review, end-users of CliniQCT or QCT Pro typically retrieve eligible CT scans into their standard PC workstation from any PACS archive (at any time), perform the hip/spine analysis on a local copy of the CT scans and create a compliant clinical report. Alternatively, radiographers may decide to send CT scans from the actual CT scanning console to the Mindways DICOM server (i.e. both ‘push’ and ‘pull’ of CT images is supported). The software connects directly with any hospital PACS infrastructure to facilitate these retrieval and send/archive steps. Mindways software features a simple graphical user interface (GUI), guiding the user through bone analysis, creation of the report, printing and, if required, exporting the results back to the PACS archive to sit alongside the CT slices for all PACS users to see; analysis takes 2–3 min. The current Slicepick module displays anterior–posterior (AP) and lateral flattened composite spine images and contains basic measurement tools for identifying and confirming vertebral fracture by morphometry. The QCT Pro measurement of spine BMD (typically L1–L3 but supported from lower thoracic to lower lumbar spine with age and sex-matched reference data) has FDA approval, as does the CTXA method for diagnosing osteoporosis. As outlined above, the ACR threshold for spinal osteoporosis <80 mg/cm^3^) is very strongly associated with prevalent and incident vertebral fracture.

Even more important than the diagnostic accuracy and utility of CTXA measurements of FN BMD are their clinical utility when imported into the FRAX tool. Thus patient-specific 10-year major and hip osteoporotic fracture risk augmented by FN BMD (the gold standard recommended by most national guidelines) can be achieved if patients first fill in the FRAX questionnaire before CT. Indeed, The FRAX tool BMD entry has a specific ‘Mindways’ category reflecting the acceptance of this way of measuring bone density by the ISCD and FRAX; inputting CTXA density to FRAX is possible in 66 countries worldwide at the time of writing. The feasibility of opportunistic screening for osteoporosis and vertebral fractures using CliniQCT and CTXA with FRAX is currently being tested in the PHOENIX study (ISCRTN 14722819, https://doi.org/10.1186/ISRCTN14722819.)

### Optasia medical

Optasia Medical specialises in software powered by ML algorithms that support the opportunistic case-finding of vertebral fracture patients. The Optasia Medical ASPIRE service out-sources the reporting of vertebral fractures visualised incidentally on CT, using a high degree of automation, combined with oversight from an in-house radiologist to improve the accuracy and efficiency of VF reporting. The service is already regulated by the CQC in the UK, and the technology has achieved CE-marking. Their technology, developed together with academic partners in University of Manchester, UK, provides a semi-automated quantitative vertebral morphometry devised from shape-based statistical modeling.^64–69^ These are used to identify and grade vertebral fractures using output measurements including vertebral height measurements and ratios and vertebral fracture classifications. In a real-world test of the software capability on CT scout views, their earlier SpineAnalyzer software, applied to CT lateral scout views, provided good-excellent agreement with the standard radiologist grading for prevalent vertebral fractures, with excellent intra and inter-reader reliability (coefficients 0.96–0.98).^67^

**Figure 2.**
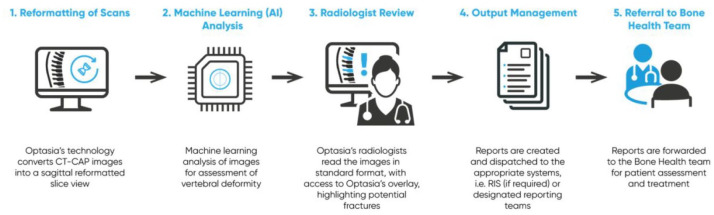
Optasia medical service provision (CQC approved). CQC, Care Quality Commission.

The ASPIRE software is designed to interface to a hospital PACS *via* a virtual machine running on a remote network. The software searches PACS for any relevant CT scans that include the spine and fulfil other criteria for example, patients >50 years of age. Identified scans are analysed and the output is reviewed by the radiologist who confirms or refutes the diagnosis, following which a report is automatically generated and returned to the requesting hospital site, the patients GP, and their local FLS or bone health team (Figure 2).

Retrospective feasibility studies involved a random sample of 1638 scans from five UK NHS Hospitals (Croydon, Cambridge, East Lancashire, Oxford and Salford).^70^ Vertebral fractures were identified in 237 patients (14.2% ± 2.0). Only 67.7% of patients with vertebral fracture identified by the service had been found in the original radiology report, and only 13.3% of patients had been referred for appropriate management. In other feasibility studies of two different NHS sites (*n* = 7103), vertebral fractures were found in 20% of cases, of which 34% had been identified in radiology reports and 5.2% had been referred for appropriate management. As a result of the study, 1205 patients were referred by the service. These data were used to change practice in Croydon, where local physicians implemented a new reporting system to alert referrers so that when incidental fractures are found on CT they undertake a bone health review according to local pathways, ensuring timely assessment and treatment as appropriate.^71^

### Zebra Medical Vision

Clinicians are keen to explore ‘point of review’ tools that alert the specialist radiologist that the CT scan they are reviewing has a prevalent vertebral fracture, ‘red flags’ for eventual future vertebral fracture or prevalent osteoporosis. Zebra Medical Vision (Zebra-Med), focuses on AI in medical imaging.^72–74^ In May 2020, their software was FDA approved for opportunistic detection in osteoporosis. Zebra-Med analyses chest and abdominal CT scans using deep neural network technology: a combination of convolutional neural network and recurrent neural network technology.^75,76^ These analyse data from the spine to analyse bone density and detect vertebral fractures. The software uses statistical and machine learning methods to identify vertebral fractures, to measure the minimal L1–L4 vertebral spine density or to emulate a lumbar spine DXA T-score. The latter DXA emulation approach is different to those listed in 4a and 4b and cannot be imputed to FRAX at present. The DXA emulation method could be run on 96.5% of CT scans in a very large cohort, whereas the method approximating L1–L4 minimal trabecular density could be run successfully on 62.3% of CT scans.^77^

In addition, Zebra-Med has released another bone health application, based on DL, to automatically identify fractures.^78^ The fracture-identifying component of Zebra’s AI1 software could be run successfully on 84.3% of CT scans in the aforementioned cohort. The software extracts a virtual sagittal section visualising the spinal mid-plane and identifies VFs using ML algorithms. It outputs the probability that the volume contains a VF, and a heat map indicating the probable location of the VF in the sagittal image. In a single-site ‘real world’ clinical implementation study involving thoracic CT scans from 1696 patients with a VF prevalence of 24%, the system achieved a sensitivity of 54%, specificity of 92% and accuracy of 83%. The radiologist or other clinician is tasked with confirming whether the algorithmic output is correct and, if so, to grade the fracture.^79^ From 48,227 individuals (51.8% women) age 50–90, the Zebra-Med algorithms applied together showed non-inferiority to basic FRAX in assessing 5-year fracture risk, and slightly better sensitivity and positive predictive value (+2.4%, +0.7% respectively). A shortcoming is that the study used only the most basic FRAX from charts rather than the online calculator to derive FRAX estimates; this is therefore based on the number of risk factors rather than the actual individually weighted risk factors.

In a different study using chest and abdominal CT scans from 1000 patients, sensitivity, specificity and accuracy were 84%, 73% and 82% respectively.^80^ Simulated T-scores for 1693 CT studies compared with DXA showed few false positives (*n* = 92) relative to true positives (*n* = 1444) but more false negatives (*n* = 212) compared with true negatives (*n* = 245.) Clinical applications have been implemented in Europe and the US and, in partnership with tele-diagnostic company Tererad Tech, have expanded Zebra’s cloud-based DL analytics engine to more than 20 countries and 150 hospitals and healthcare organisations in India, Africa and Asia.

### CT measurements of Hounsfield units

While there are many research studies evaluating CT HU for osteoporosis screening applications, the HU thresholds for consistent application across devices vary both with device and with protocol so it is not considered feasible to undertake a calibration exercise for each combination of device and protocol, meaning that such methods have not achieved clinical adoption and are unlikely to ever fulfil ‘approved technology’ status (see above for definitions). They are not dealt with further in this review.

## Cost-effectiveness, futility and acceptability studies

For the implementation of opportunistic screening of CT for osteoporosis in a healthcare setting, understanding of its cost-effectiveness is vital, especially given the amount of work generated downstream for FLS and prescribers. It is also important to know in which patients screening would be futile. This is particularly true for opportunistic evaluation of CT; many routinely acquired CT scans are for patients with cancer or cancer-monitoring in whom mortality is higher than the general public. Indeed, the ancillary finding of a vertebral fracture in CT reduces survival markedly; from around 60% 4-year survival to about 30% 4-year survival (in adults 75 years or older undergoing chest CT)^81^. Here, there is a large research gap.

There have been only a small number of cost-effectiveness studies published. In a state-transition simulation study of a hypothetical cohort of 1 million post-menopausal women age >55 years, a screening programme of combined DXA and QCT performed at age 55 years with subsequent QCT every 5 years, was found to be most cost effective [$2000 per quality-adjusted life year (QALY)].^24^ With this strategy, there was a 12.8% lifetime hip fracture risk, compared with 18.7% with no screening and 15.8% with DXA alone. Favourable outcomes were also seen for wrist and vertebral fractures. However, this study is specific to a US Healthcare system model, and focused mostly on White American women. In an earlier, separate analysis, Viceconti calculated that BCT could be cost effective in the UK at $14,656 per QALY,^47^ if offered at a fee of $100 per patient in addition to payment for a dedicated CT examination.^82^ Neither of these studies consider an opportunistic approach, and do not study men.

A more recent analysis focussed on a one-time ancillary BCT offered only to patients already undergoing abdominal CT, and who had not had a recent DXA.^83^ Researchers used a one-time biomechanical CT test (VirtuOst) to assess the accuracy and cost-effectiveness of this strategy in male and female patients aged >65 years in a hypothetical cohort of 1000 patients who underwent either this BCT approach or usual care using DXA or no screening. The BCT approach proved more cost-effective and clinically beneficial. Using the biomechanical CT strategy, 90% of women were screened and 21% tested positive for osteoporosis. Using DXA, 37.4% were screened and 12.5% tested positive for osteoporosis. For women, when using the no-screening model as a reference point, biomechanical CT prevented 5.5 hip fractures while DXA prevented 2.4 hip fractures. For men, biomechanical CT prevented 2 hip fractures and DXA prevented 0.2 hip fractures. When screening was restricted to patients at a 2-fold higher risk for hip fracture, prevented hip fractures also increased 2-fold with biomechanical CT, with a proportional increase in cost savings. These studies show promising results; however, more data specific to different healthcare systems and populations are crucial in the integration of these technologies into the healthcare setting. Finally, there is no data published describing patient attitudes to being offered ‘opportunistic’ screening. While there are general support from the osteoporosis patient community, work is needed to identify concerns and expectations among CT attenders.

## Barriers for implementation of novel osteoporosis screening technologies and possible solutions

### Non-adoption, adoption or abandonment of osteoporosis screening technologies and the challenges to scale-up, spread and sustain such technologies in healthcare organisations and systems

It takes on average 17 years to incorporate research discoveries into the practice of healthcare providers.^84^ A thorough review of this topic is beyond the scope of this rapid evidence review; therefore, we include a few highlights and key themes relevant to embedding existing technologies into the NHS. Greenhalgh *et al.* developed an evidence-based framework for studying the non-adoption, adoption or abandonment of technologies and the challenges to scale-up, spread, and sustain such technologies in healthcare organisations and systems (abbreviated NASSS).^85,86^ The NASSS framework includes seven domains: the condition/illness, the technology, the value proposition, the adopters, the organisation(s), the wider context and changes over time. Each domain can be rated from simple to complex, with more complex projects being associated with higher failure rates. The NASSS framework can be applied to technologies in health and care either prospectively, to guide design and implementation, or retrospectively, to learn from failure. A diverse range of technology-supported programmes has been tested using this framework. Failure is often linked with complexity across multiple NASSS domains, and 10 principles have been highlighted to help manage and minimise this complexity. Table 2 shows the application of these principles to the current osteoporosis challenge. Opportunistic screening for osteoporosis and vertebral fractures in CT comes up against the four well-recognised barriers to implementation of technology in the NHS. First, against poor communication and connectivity, which slows innovation across individuals and organisations due to the fragmented structure of UK health services. Second, against lack of evaluation by NICE of the complex new technology. Third, against a lack of funding to take technologies forward for implementation at scale, even after successful pilots. Finally, even well designed innovations require system changes that the NHS is simply unable to afford the time, money and staff to implement, despite clear evidence that these changes would bring major benefits in the long run. An Institute of Public Policy Research report concluded that whilst barriers can vary between different innovations, a number of common problems exist across most innovations, namely: complexity, culture and money.^87^ Several organisations and reports have highlighted challenges in implementing novel technologies in the NHS and provided some guidance on how these can be overcome. These include The Nuffield Trust, The Kings Fund and the Institute for Public Policy Research.^88,89^

**Table 2.** The 10 NASSS principles applied to opportunistic analysis for osteoporosis using clinical CT (Greenhalgh).^90^

1. Strengthen program leadership across academic and commercial research, NHS radiology, FLS, IT, metabolic bone, patient and public involvement, NHS procurement and management departments
2. Develop a vision for National opportunistic screening of CT scans for osteoporosis and fractures
3. Nurture key relationships between software developers, designers, vendors, image analysis providers, NHS X, CCGs, NIHR, RCR, Society of Radiographers, image exchange portal, ROS, ISCD and other essential stakeholders
4. Develop champions through the national Academy initiatives and encourage them to problem solve local problems creatively
5. Make resources available *via* the academy and other funding organisations for creative individuals/teams to use for generating solutions to local challenges to implement image analysis
6. Capture data on progress and feedback to leadership, teams and individuals
7. Acknowledge and address concerns of frontline NHS staff from idea to implementation
8. Work with intended users to co-design practice-ready imaging technologies and FLS integration
9. Control scope of the project, for example, concentrating initially on moderate- severe vertebral fractures
10. Address regulatory and policy barriers *via* CE marking, ISO certification, FDA approval, ISCD

CCG, clinical commissioning groups; CT, computed tomography; FDA, United States Food and Drug Administration; FLS, fracture liaison service; ISCD, International Society for Clinical Densitometry; ISO, International Organization for Standardization; IT, information technology; NHS, National Health Service; NIHR, National Institute of Health Research; RCR, Royal College of Radiologists; ROS, Royal Osteoporosis Society.

Several initiatives and organisations exist that try to improve the process. The NHS Accelerated Access Collaboration, NHS Innovation Accelerator and its associated programmes support fast-track of innovations from idea to adoption and spread; evaluation of this organisation has shown effectiveness in scale-up and spread of innovations.^87,91–96^ In April 2020, in the NHS Long Term Plan, a MedTech Funding Mandate was introduced as part of the wider strategy to accelerate the uptake of NICE-approved cost-saving MedTech products in the NHS.^97–99^ Evidence shows that nationally managed schemes resulted in a more rapid and complete uptake compared with devices that were not part of a national programme.^100^ In February 2019, NHSX was established as a government unit that is responsible for setting consistent national policy and developing best practice for technology, digital services and data throughout the NHS.^101,102^ NHSX is actively looking at screening programmes for high-risk populations, but it is currently unclear whether or how this unit’s work would be relevant to this technology’s implementation.

### How osteoporosis screening from clinical CT could follow a successful pathfinding CT software technology solution into routine NHS practice: HeartFlow FFRCT

An example of NHS software technology adoption is HeartFlow FFRCT – a technology that was recommended by NICE under its medical technologies guidance work stream (MTAG).^103^ HeartFlowFFR is used to estimate fractional flow reserve from CT coronary angiography, and may avoid the need for invasive coronary angiography in patients with stable, recent onset chest pain. Draft recommendations based on all the evidence presented in the support of the technology were given, considering key clinical outcomes. NICE considered a total of over 69 studies comprising diagnostic accuracy, clinical effectiveness and cost-evidence. It is notable that this represents far more evidence than is available for the osteoporosis technologies. Whilst HeartFlowFFR was selected by the MTAG committee in December 2014, the final guidance was not published until February 2017 (27 months) demonstrating the long timescales often involved.

## Discussion: areas for further research and development

Ancillary screening of CT data for osteoporosis and vertebral fractures is well supported by numerous academic papers focussed on software development and successful use in clinical practice. Various tools can now provide a rapid and reproducible screening method for osteoporosis and previously unidentified fractures. However, there are areas where further research is needed in order to address evidence gaps. It is currently unclear which patient groups should be included in opportunistic screening. It could be used exclusively in older adults, or also include other high-risk groups. A large proportion of routine CT attenders have specific co-morbidities, such as cancer, in comparison with the general population. Thus, while they have a higher unmet osteoporosis burden, the effects of screening, treatment and survival in these attenders needs to be understood in order to ascertain its clinical impact and cost-effectiveness.

It is yet to be determined how opportunistic CT imaging could be clinically integrated with current diagnostic methods. Determining whether it would be used in addition to, or instead of DXA, and for screening and/or definitive diagnosis, remains to be established. Further data will be required on which site(s) should be primarily used in opportunistic CT screening; for example, regions within the lumbar spine or hip and, if so, which sub-regions. The additional value of measuring hip and spine bone strength with CT FEA (to diagnose and treat patients on the basis of FBS) over simpler QCT methods needs to be quantitated. There are also no head-to-head studies providing comparative data that assess whether technologies that detect fracture are more effective than those assessing bone density/ bone strength, or comparing these technologies with any other methods of opportunistic screening.

Further understanding of the technology itself will be key to its widespread implementation. Each of the different calibration techniques has advantages and pitfalls; and additional research is necessary to characterise the sources of variation between scans using each calibration technique. In addition, the exact effect of IV contrast on the accuracy of the data is not yet known.

There are several service delivery issues. Should the services be standalone outside the NHS, embedded as ‘point of care’ or near ‘point of care’ tools and when should CT data be ‘sent’ for screening? A key emerging issue is the ability for healthcare providers to manage the higher workload resulting from increased case-finding. FLS and other healthcare providers could potentially be required to consult, administer treatment, follow up and monitor vastly increased numbers of affected patients. Local systems for service delivery would need to be established. These include logistics of how relevant diagnostic images would be stored, transmitted to healthcare providers (HCPs) and FLS teams, and how follow-up measurements, for instance, with DXA, would be comparable for the purposes of monitoring or drug-cessation.

## Conclusion

Osteoporosis imposes a significant public health impact, as well as cost burden, and is increasing in prevalence. It remains under-diagnosed and under-treated. There is evidence from the literature to support multiple technologies using opportunistic screening of CT scans done for other indications, which could increase the rates of diagnosis, and therefore treatment to prevent fractures. There are still areas where further research is needed. However several barriers remain to the implementation of technologies into healthcare systems; encompassing problems with culture, complexity and funding. With further research and the use of new and existing initiatives, there may be opportunities for the implementation of these technologies into clinical practice.
